# Virulence Regulator EspR of *Mycobacterium tuberculosis* Is a Nucleoid-Associated Protein

**DOI:** 10.1371/journal.ppat.1002621

**Published:** 2012-03-29

**Authors:** Benjamin Blasco, Jeffrey M. Chen, Ruben Hartkoorn, Claudia Sala, Swapna Uplekar, Jacques Rougemont, Florence Pojer, Stewart T. Cole

**Affiliations:** 1 Global Health Institute, Ecole Polytechnique Fédérale de Lausanne (EPFL), Lausanne, Switzerland; 2 Swiss Institute of Bioinformatics, Lausanne, Switzerland; Harvard School of Public Health, United States of America

## Abstract

The principal virulence determinant of *Mycobacterium tuberculosis* (*Mtb*), the ESX-1 protein secretion system, is positively controlled at the transcriptional level by EspR. Depletion of EspR reportedly affects a small number of genes, both positively or negatively, including a key ESX-1 component, the *espACD* operon. EspR is also thought to be an ESX-1 substrate. Using EspR-specific antibodies in ChIP-Seq experiments (chromatin immunoprecipitation followed by ultra-high throughput DNA sequencing) we show that EspR binds to at least 165 loci on the *Mtb* genome. Included in the EspR regulon are genes encoding not only EspA, but also EspR itself, the ESX-2 and ESX-5 systems, a host of diverse cell wall functions, such as production of the complex lipid PDIM (phenolthiocerol dimycocerosate) and the PE/PPE cell-surface proteins. EspR binding sites are not restricted to promoter regions and can be clustered. This suggests that rather than functioning as a classical regulatory protein EspR acts globally as a nucleoid-associated protein capable of long-range interactions consistent with a recently established structural model. EspR expression was shown to be growth phase-dependent, peaking in the stationary phase. Overexpression in *Mtb* strain H37Rv revealed that EspR influences target gene expression both positively or negatively leading to growth arrest. At no stage was EspR secreted into the culture filtrate. Thus, rather than serving as a specific activator of a virulence locus, EspR is a novel nucleoid-associated protein, with both architectural and regulatory roles, that impacts cell wall functions and pathogenesis through multiple genes.

## Author Summary

A major infection mechanism employed by the causative agent of tuberculosis, *Mycobacterium tuberculosis (Mtb)*, is the ESX-1 secretion system. It has been postulated that the DNA-binding protein EspR controls the virulence of *Mtb* by specifically regulating expression of the exported EspA protein, which is required for ESX-1 to function. Previous structural studies indicated that EspR forms dimers capable of multimerizing on DNA and forming loop structures, thus bringing together otherwise distant chromosomal regions. Such characteristics are reminiscent of nucleoid-associated proteins (NAPs), the histone equivalent in bacteria. Here we use ChIP-Seq technology to map EspR binding sites on the *Mtb* chromosome in living bacterial cells. Genome-wide analysis of EspR identified hundreds of binding-sites, with almost equal inter- and intra-genic distribution, and mostly found in proximity to genes associated with cell wall function. We validated a subset of EspR-binding sites experimentally and identified a consensus motif required for optimal binding affinity. Moreover, our study reveals that EspR expression varies with bacterial growth and that intracellular levels are not linked to EspR secretion. These findings corroborate the NAP nature of EspR and its dual roles, architectural and regulatory, that impact the *Mtb* chromosome and pathogenesis globally rather than the ESX-1 loci specifically.

## Introduction

Details of the genetic control mechanisms governing the pathogenicity of the etiological agent of human tuberculosis are starting to emerge [1]. It has been postulated that the DNA-binding protein EspR [2] controls the virulence of *Mycobacterium tuberculosis* (*Mtb*) by specifically regulating expression of EspA, an exported protein [3], [4], which is required for the ESX-1 system to function normally. There are five genetically related ESX-systems in *Mtb* but functional information is scarce for all of them, although ESX-1 is by far the most studied [5], [6]. ESX-1 is widely considered to be the principal virulence determinant of *Mtb* since it secretes the EsxAB (ESAT-6 and CFP10) proteins and ESX-1 secretion-associated proteins (Esps) [7]. Although mechanistic details are limited, some of these secreted proteins act as effector proteins that perturb host cell activities, permeabilize the phagosomal membrane and allow the tubercle bacillus to escape into the cytoplasm [6], [8], [9].

Structural studies revealed that EspR is a homodimer with two domains: an N-terminal DNA-binding domain with a helix-turn-helix (hth) motif and C-terminal domain that mediates dimerization [10], [11]. Removal of 10 amino-acid residues from the C-terminus, as in the EspRΔ10 protein, does not affect DNA-binding activity but prevents dimerization and ablates activation of the *espACD* locus [2], [10]. A model has been proposed, based on the results of co-crystallization with DNA and molecular dynamic simulations, wherein EspR employs an atypical DNA-recognition mechanism involving a dimer of dimers. Since, for sterical reasons, only one hth from each dimer is capable of inserting into the major groove of DNA at a given binding site, the second hth of each dimer remains free to act at other binding sites [10]. Consequently, dimers can dimerize then multimerize and recognize distal DNA binding sites in a cooperative manner as has been observed by atomic force microscopy (AFM) of EspR-nucleoprotein complexes at the *espA* locus, where DNA bending and bridging resulted in loop formation [10]. This behavior is characteristic of nucleoid-associated proteins (NAPs) rather than that of a classical gene activator protein [12], [13]. NAPs are the bacterial equivalent of histones that organize the chromosome and act by stabilizing long-range structures in the genome through cooperative binding to multiple sites. This results in modulation of the accessibility of free DNA to the transcription machinery via the state of DNA compaction. NAPs thus function in a quite different manner to transcriptional activators, which typically recognize a limited number of sites in the genome and promote transcription through direct interaction with RNA polymerase.

To test the hypothesis that EspR might be a NAP and to gain more insight into the extent of EspR-mediated regulation in living mycobacteria, ChIP-Seq analysis of the *Mtb* H37Rv genome was performed. Unlike cDNA hybridization to micro-arrays, ChIP-Seq generates quantitative information with single nucleotide resolution. The complete repertoire of EspR-binding sites across the chromosome was established with the majority of the gene targets appearing to impact cell wall function generally rather than ESX-1 expression in particular. The ChIP-Seq findings were confirmed independently by ChIP followed by quantitative real-time PCR (ChIP-qPCR), by gel shift and DNase I footprinting assays, as well as by transcript analysis, and a binding motif was deduced using bioinformatics. Finally, the expression levels of EspR and its subcellular localization were monitored and quantified during the growth cycle. Altogether, these data provide a genome-wide view of EspR regulatory functions and place EspR firmly in the NAP family.

## Results

### Genome-wide analysis of the EspR regulon

**Figure 1:**
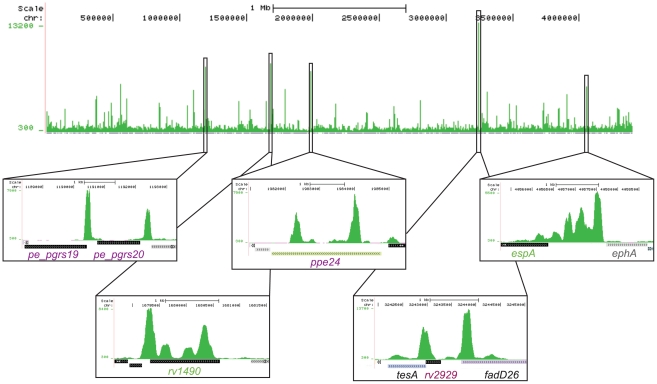
Genome-wide mapping of EspR binding sites. UCSC Genome Browser (http://genome.ucsc.edu) view of EspR binding across the *Mtb* genome as determined by ChIP-Seq. Peak height (y-axis) indicates the sequencing read depth at each genomic position (x-axis). Inset boxes show major EspR binding sites identified over (from left to right) the *pe_pgrs19* and *pe_pgrs20* genes, the *rv1490* gene, the *ppe24* gene, the *rv2929* and *fadD26* genes from the PDIM/PGL locus and the *espACD* operon promoter region.

**Figure 2:**
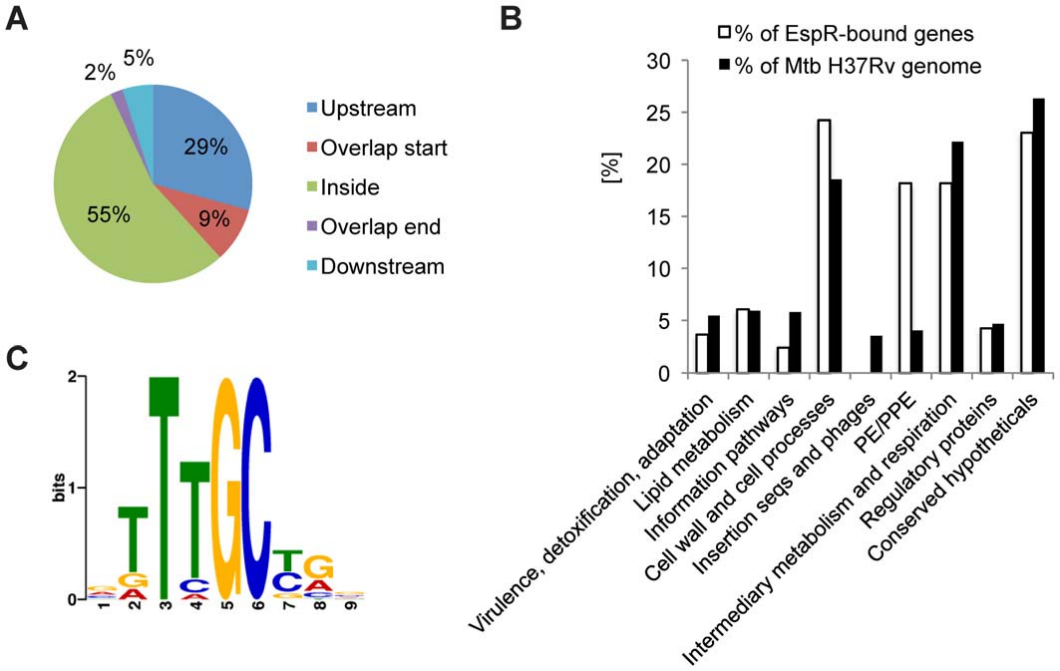
Binding of EspR to the *Mtb* chromosome. (**A**) Pie chart of EspR-binding peaks annotated relatively to the nearest gene translation start using the *ChIPpeakAnno* package [38]. (**B**) Bar chart displaying the percentage of genes of the EspR regulon compared to the *Mtb* H37Rv genome belonging to functional categories defined in the TubercuList database (http://tuberculist.epfl.ch/). (**C**) Most significant motif derived from ChIP-Seq binding sequences returned by the MEME tool [19]. The height of each letter represents the relative frequency of each base at different positions in the consensus.

We investigated EspR-binding to the chromosome of *Mtb* strain H37Rv during exponential growth by ChIP-Seq, chromatin immunoprecipitation followed by ultra-high throughput DNA sequencing [14]. Sequence reads obtained from two independent ChIP-Seq experiments using EspR-specific antibodies were mapped to the *Mtb* H37Rv genome. Based on the peak detection criteria, we identified 165 enriched loci harboring 582 EspR-binding peaks (Fig. 1, Table S2), that were enriched by >1.5-fold and these were not present in ChIP-Seq datasets from control experiments conducted without antibody or with unrelated antibodies (data not shown). These 165 loci occurred across the genome (Fig. 1 and Fig. 2A) and were sited both in intergenic regions (45%) and within genes (55%) implying that EspR is not a classical transcriptional regulator.

Diverse functions are encoded by genes where EspR bound upstream and classification by functional category reveals over-representation of cell wall/cell processes and the surface-exposed PE/PPE proteins (http://tuberculist.epfl.ch/; Fig. 2B). Internal sites were found within AT-rich genes encoding proteins belonging to the PPE family (Fig. 1), like *ppe24* (*rv1753c*), and some of these, such as *ppe58* (*rv3426*), also bind EspR at their 5′-ends. Binding sites were present within genes that are thought to have been acquired by horizontal transfer [15] like the *rv0986-rv0989c* region.

**Figure 3:**
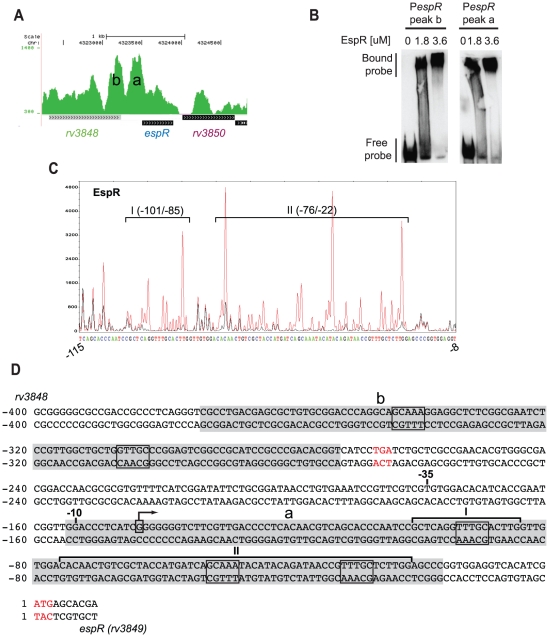
EspR autoregulation. (**A**) Pattern of EspR ChIP-Seq peaks (denominated peaks **a** and **b**) identified upstream of the *espR* start codon. (**B**) EMSA showing concentration-dependent EspR binding of DNA fragments covering the center sequences of peaks **a** and **b** shown in (A). (**C**) DNase I footprint from binding of EspR to peak **a** of *espR*. Red and black peaks represent DNA without or with 10 µM EspR, respectively. Both reactions were partially digested with DNase I and analysed by capillary electrophoresis in a genetic analyser (Applied Biosystems 3130xl). The corresponding nucleotide sequence is shown below. Regions I and II protected from DNase I digestion by EspR are denoted by black brackets and positions relative to the translational start are indicated. (**D**) Analysis of the *espR* promoter. The 5′ end of the transcript was determined by 5′ RACE using RNA extracted from *Mtb* H37Rv at mid-log phase. Translational stop of *rv3848* and translational start of *espR* are highlighted in red with +1 corresponding to the first base of the *espR* open reading frame. Transcriptional start mapped by 5′ RACE is boxed and indicated by a bent arrow. −10 and −35 positions for putative sigma factor binding sites are indicated. ChIP-Seq peak sequences are highlighted in gray, EspR consensus motifs framed and sites protected from DNase I cleavage bracketed as in (C).

A survey of the ten top scoring peaks (Table 1) highlighted the major EspR-binding gene targets. Two of the top three sites (Fig. 1) occurred at a locus encoding an enzyme system that produces the complex lipids phthiocerol dimycocerosate (PDIM) and phenolic glycolipid (PGL) [16], [17]. The second highest scoring site overlaps the translational start of *rv1490*, which encodes a membrane protein of unknown function, and this was followed by three other peaks of lower intensity, separated by ∼300–400 bp, spread across *rv1490* (Fig. 1). The fourth and eighth highest scoring sites affect two genes, *pe-pgrs19* and *pe-pgrs20* [18], encoding mycobacteria-restricted PE_PGRS proteins, while the *espACD* locus, which is preceded by three EspR-binding sites (Fig. 1), occurred in the fifth position of the top ten ChIP-Seq hits (Table 1). The ninth peak is sited in the intergenic region between *lipF* (*rv3487c*), encoding a lipid esterase, and *rv3488*, whereas the last peak of the top ten ChIP-Seq list was found at the 3′-end of *fadB2* (*rv0468*) encoding a beta-hydroxybutyryl-CoA dehydrogenase and upstream of *umaA* (*rv0469*) coding for a mycolic acid synthase. EspR binds to multiple sites in the ESX-1, ESX-2 and ESX-5 loci (Fig. S2), as well as to two sites upstream of its own gene (Fig. 3), thus implying autogenous control. Taken together, these data suggest that EspR may be involved in regulating cell wall function.

**Table 1:** Top 10 EspR binding loci from ChIP-Seq.

Gene	Position	Gene description	Functional category
*rv2930*	inside	FadD26, fatty-acid-CoA synthetase, PDIM production	lipid metabolism
*rv1490*	overlaps start	Rv1490, probable membrane protein	cell wall and cell processes
*rv2929*	overlaps start	Rv2929, PDIM production	conserved hypotheticals
*rv1067c*	overlaps start	PE_PGRS family protein, PE_PGRS19	PE/PPE
*rv3616c*	upstream	EspA, ESX-1 secretion-associated protein A	cell wall and cell processes
*rv1753c*	inside	PPE family protein, PPE24	PE/PPE
*rv0986*	inside	ATP-binding protein of ABC transporter	cell wall and cell processes
*rv1068c*	upstream	PE-PGRS family protein, PE_PGRS20	PE/PPE
*rv3487c*	upstream	LipF, probable esterase/lipase	lipid metabolism
*rv0469*	upstream	UmaA, Mycolic methyltransferase A 1	lipid metabolism

### Consensus sequence

An EspR consensus sequence was identified in the ChIP-Seq peaks using the MEME suite [19] and the binding motif deduced (Fig. 2C). FIMO (Find Individual Motif Occurrences) detected 736 occurrences of this motif (*p*-value≤0.001) distributed among 80% of the EspR peak sequences analyzed. Of these motifs, 59% were localized within open reading frames (ORF). The entire *Mtb* genome sequence was searched for potential EspR-binding sites using the TTTGC[TC][GA] consensus sequence and 199 putative intergenic and 827 intragenic sites were identified, of which 163 (43 intergenic and 120 intragenic) correspond to known ChIP-Seq peak sites. Further experimental support for the consensus sequence is available from footprinting studies of the *espA* [10] and *espR* promoter regions (see below), which revealed EspR protection from DNase I digestion at sites comprising at least one TTTGC-like motif.

### Confirmation of *in vivo* EspR binding

To obtain independent confirmation for selected parts of the *in vivo* dataset, we initially focused on the EspR-dependent *espACD* operon [2]. Our previous *in vitro* work revealed two EspR binding sites separated by 19 bp and located between 506 and 444 bp upstream of *espACD* [10], consistent with the presence of a ChIP-Seq peak in this region (Fig. 1). On closer inspection, two additional major peaks of EspR-enrichment were found further upstream of *espA* (centered between −857 bp and −695 bp and between −1214 bp and −1113 bp, respectively). While this work was in progress, another report of the presence of two additional sites upstream of *espACD* appeared [11]. The existence of these sites also corroborates results we obtained previously using AFM to visualize nucleoprotein complexes of EspR and a 1360 bp *espACD* promoter fragment [10]. AFM revealed loop structures stabilized by multiple EspR dimer of dimers suggesting the presence of several distant EspR binding sites in the *espACD* upstream region. The 5′-end of the *espA* mRNA was located 66 bp upstream of the translation start codon using 5′ RACE (Fig. S3). Consequently, the nearest EspR binding site is positioned over 300 bp upstream of the promoter.

To further validate EspR-binding peaks, with varying degrees of enrichment, we performed ChIP followed by quantitative PCR on 11 selected sites (four located within intergenic regions, three within ORFs and three overlapping a translational start) and two non-peak regions (within *rv0888* and *sigA* ORF) as controls. All of the selected EspR-binding regions exhibited enrichment comparable to that observed from ChIP-Seq analysis (Fig. S4), thus confirming that all peaks were genuine EspR-targets.

To obtain further confirmation of the *in vivo* EspR binding sites, we performed electrophoretic mobility shift assays (EMSAs) using ∼100 bp DNA sequences covering the top five binding sites (Fig. S5 and Table 1) and a DNA fragment of the same size from within the *espA* ORF as a negative control. EspR was shown to bind to all five sites in a concentration dependent manner, while the negative control fragment remained unbound at an equal protein concentration. However, clear differences in affinity between the fragments were visible. For example, the top-scoring *fadD26* peak bound EspR less strongly compared to the four others suggesting that other determinants, like long-range protein-protein or protein-DNA interactions, could contribute to the high-affinity binding observed *in vivo*.

### Autogenous regulation at the *espR* promoter

The presence of twin peaks upstream of *espR* (**a**, **b** in Fig. 3A) is suggestive of autoregulation. To test this possibility, we performed EMSA, DNase I footprinting and 5′ RACE analysis of the *espR* promoter region. Peaks **a** and **b** were both shown to bind EspR using EMSA (Fig. 3B). Two regions within peak **a** were protected from DNase I by EspR; region I, covering 17 bp, and region II, 55 bp-long, are situated 101 and 76 bp upstream of the *espR* translational start codon, respectively (Fig. 3C and 3D). This binding pattern is reminiscent of that described previously at the *espA* promoter [10]. When incubated with the dimerization deficient EspRΔ10 protein, only part of region II and none of region I was protected (IIa_Δ10_, 14 bp and IIb_Δ10_, 12 bp, see Fig. S6). This implies that oligomerization enables cooperative binding between multiple EspR dimers, leading to the formation of higher-order oligomers. The zone protected by EspRΔ10 contains an inverted repeat of two consensus motifs: CAGCAAA<16>TTTGCTC.

5′ RACE analysis was employed to localize the *espR* promoter using RNA extracted from *Mtb* H37Rv grown to mid-log phase. The *espR* transcript starts with a poly-G (7) sequence 144 bp upstream of the translational start codon. The promoter is therefore situated in a region between peaks **a** and **b** so simultaneous occupation by EspR of both the **a** and **b** sites might form a repression loop. Expression data presented below indicate a negative effect of EspR on its own transcription.

### Target gene regulation on EspR binding

**Figure 4:**
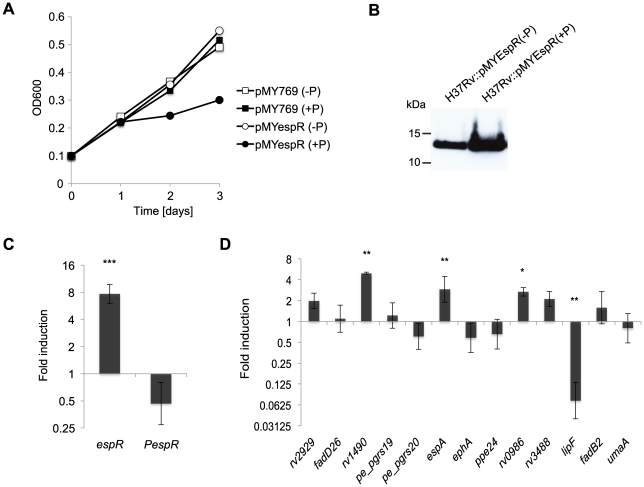
Gene expression associated with EspR binding. (**A**) Growth of H37Rv::pMYespR without (−P) or with (+P) pristinamycin IA. Growth was monitored at the designated time points measuring OD_600_. (**B**) Immunoblot analysis of EspR expression in the absence (−P) or presence (+P) of pristinamycin IA after 3 days growth using rat polyclonal antibodies specific for EspR. Equivalent amounts of total protein lysates were loaded. (**C**, **D**) Quantitative RT-PCR analysis of relative mRNA levels extracted from H37Rv::pMYespR cells treated with 0 µg/ml or 2 µg/ml of pristinamycin using primers specific for the following regions: (**C**) the *espR* coding region (annealing to both endogenous and vector copies of *espR*) or to the 5′-UTR region of *espR* (*PespR*) (specific to endogenous *espR*). (**D**) The coding regions of genes related to the top 10 EspR binding peaks as obtained in ChIP-Seq experiments (see Table 1). Relative gene expression was normalized against *sigA* and displayed as fold-induction (log-2 scale) relative to the untreated sample (0 µg/ml pristinamycin). Shown are the mean ± s.d. of a minimum of duplicate measurements from the average of three independent experiments. Statistical significance was evaluated using Students T-test. * indicates P<0.05, ** P<0.01 and *** P<0.001.

To confirm the prediction that binding of EspR directly affects target gene expression, we exploited a pristinamycin-inducible system [20], [21] to overexpress *espR* conditionally in *Mtb* (strain H37Rv::pMYespR; Fig. 4). Compared to the controls, it is noteworthy that *espR* over-expression significantly decreased growth after 24 h (Fig. 4A) while *espR* transcript and EspR protein levels were found to be ∼8-fold and ∼3-fold higher than in the control after 72 h, respectively (Figs. 4C and 4B). When the relative amounts of target transcripts in untreated and pristinamycin IA-treated H37Rv::pMYespR cells were measured by quantitative RT-PCR, significantly increased transcript levels were detected for *rv1490*, *espA*, and the ABC-transporter *rv0986* (Fig. 4D). Conversely, repression of *lipF* transcription was also observed upon EspR overexpression, whereas transcription of some target genes appeared unchanged (Fig. 4D). Using a discriminatory RT-PCR assay it was possible to measure the impact of EspR overproduction on expression of the chromosomal copy of *espR* and, again, this appeared to act negatively (Fig. 4C).

The combined findings suggest that EspR is capable of both positive and negative transcriptional regulation. Moreover, the inability to observe direct EspR-dependent regulation at some major EspR binding sites suggests that EspR has no or little effect on these genes in the conditions tested or that other regulators counter-balance the effect of increased EspR levels.

### Growth phase dependent expression of EspR

**Figure 5:**
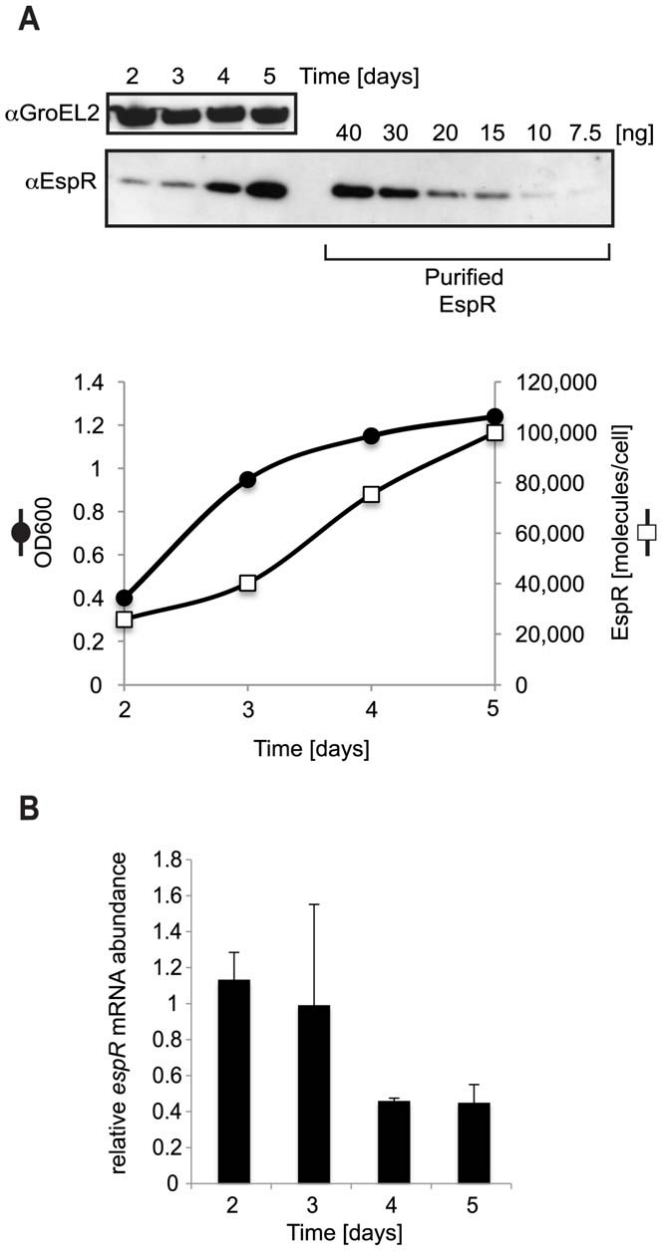
Growth phase-dependent variation of EspR intracellular levels. Time course analysis of *Mtb* H37Rv at the following phases of growth: early log (day 2), mid-log (day 3) and stationary (days 4 and 5). (**A**) Immunoblot analysis of cell lysates (CL) from equivalent amount of cells (approx. 2*10∧7). For quantification purposes, the indicated amounts of purified EspR were electroblotted onto the same membrane as cell lysates. GroEL2 is used as a loading control and corresponding Western blot signals are used to normalize small loading discrepancies. The number of EspR molecules per cell was estimated from Western blot analysis correlated to OD_600_ measurements, assuming that OD_600_ 0.2 = 10^8^ cells [40]. The results are plotted in the bottom chart, with solid circles representing optical density at 600 nm (OD_600_) while open squares represent the total number of EspR molecules per cell from one representative of four experiments. (**B**) Relative *espR* transcript amounts measured in total RNA harvested at the different time points by quantitative RT-PCR and normalized to *sigA* expression. The means and standard deviations of triplicate measurements are shown from two experiments.

It has been proposed that intracellular levels of EspR are regulated via its secretion by the ESX-1 system and that blocking EspR secretion results in enhanced EspR-mediated transcriptional effects [2]. This suggested that the intracellular requirements for EspR could change during the growth phase of *Mtb* since the secretion of other ESX-1 substrates, such as EsxA (ESAT-6), is known to occur early in the growth cycle. To determine whether levels were constant or variable during growth and to estimate the number of EspR molecules per cell, kinetic experiments were performed. We monitored EspR protein levels by quantitative Western blotting at different time points corresponding to the early-log (day 2), mid-log (day 3) and stationary (days 4 and 5) phases of growth. Analysis of equivalent cell numbers showed that the intracellular concentration of EspR increases throughout the bacterial growth cycle, ranging from ∼20,000 molecules at early log-phase (day 2) to ∼100,000 molecules per cell at stationary phase (day 5) (Fig. 5A). Peak cell concentration of EspR in stationary phase is consistent with the growth arrest observed upon its premature induction (Fig. 4A). To determine if this peak was due to protein accumulation or to increased expression of the *espR* gene, we performed quantitative RT-PCR on RNA samples isolated from cells at different time points (Fig. 5B). Interestingly, throughout the growth cycle, the levels of *espR* mRNA varied in a manner inversely proportional to the amounts of EspR protein, suggesting that EspR stably accumulates in the bacteria while autorepression may limit its own gene expression at late time points.

### EspR is not secreted

**Figure 6:**
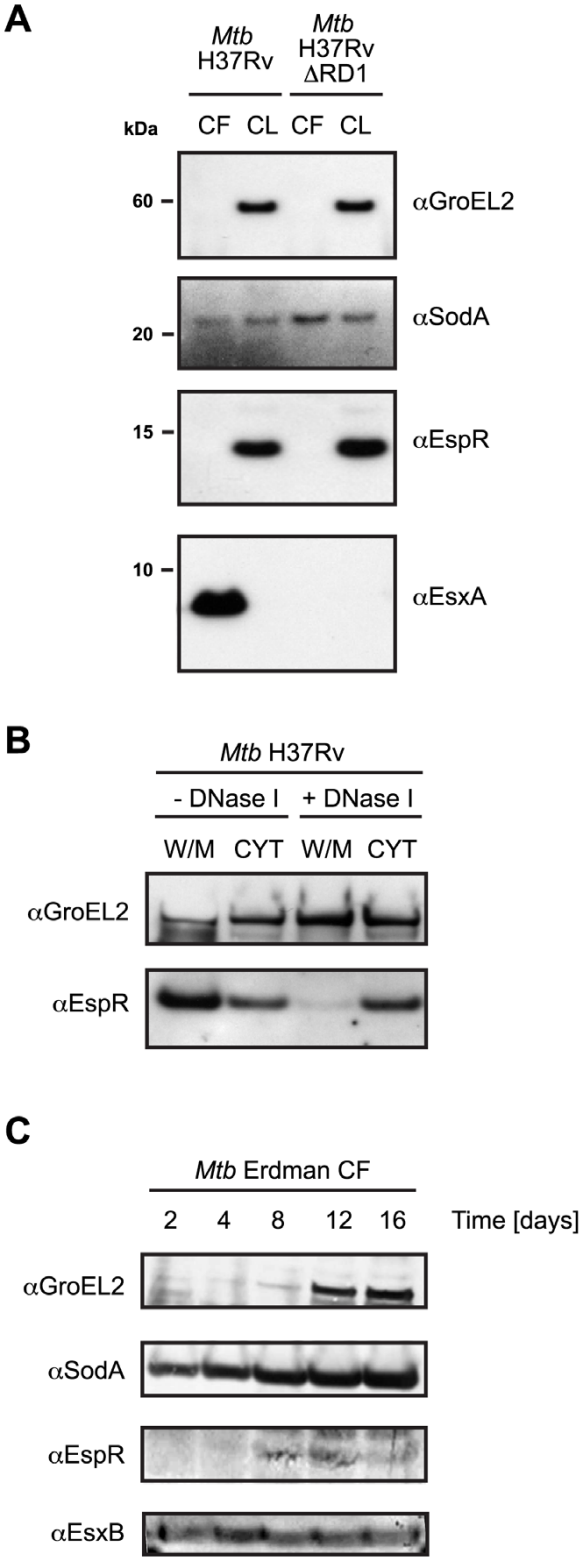
EspR is not a secreted protein. (**A**) Immunoblot analysis of 10 µg of culture filtrate (CF) and 5 µg of cell lysates (CL) of wild-type H37Rv (left) and H37RvΔRD1 (right) strains grown for 4 days after transfer into Sauton's medium without Tween-80. GroEL2 was used as a control for autolysis, SodA as a loading control for CF and CL samples and EsxA as a control for ESX-1-dependent secretion. (**B**) Immunoblot analysis of H37Rv CL shown in (**A**) fractionated into cell wall/cell membrane (W/M) and cytosolic (CYT) components by ultracentrifugation. GroEL2 was used as a loading control. (**C**) Immunoblot analysis of Erdman CF (20 µg) for each of the time points indicated.

To determine if the low intracellular levels of EspR observed at the early and mid-log phases of growth were due to intensive EspR secretion, we measured intra- and extra-cellular levels of EspR from strains *Mtb* H37Rv and *Mtb* H37RvΔRD1 cultured in Sauton's medium to mid-log phase. Under these conditions, we were unable to detect EspR among the culture filtrate (CF) proteins in either case, whereas EsxA was present in the CF of *Mtb* H37Rv, as expected, but not in CF from the ESX-1 mutant H37RvΔRD1 that lacks *esxA* among other genes (Fig. 6A).

To investigate whether EspR was exported from the cytosol but retained in the cell envelope, whole cell lysate (CL) was fractionated by ultracentrifugation into the cell wall/cell membrane (W/M) and cytosolic (CYT) components. Since the chromosome is known to be attached to the plasma membrane [22], half of the samples were treated with DNase I. EspR was detected in both of the untreated fractions but was mainly in the cytosol after DNase I treatment (Fig. 6B). Since previous studies were performed with the Erdman strain of *Mtb*, this provided a possible explanation for the localization discrepancy. Consequently, we repeated the experiment with the *Mtb* Erdman strain and the ESX-1 mutant *Mtb* Erdman 36–72 that fails to secrete EsxA [23]. Again, EspR was below the level of detection in the CF of either strain, whereas EsxA appeared in the CF of *Mtb* Erdman (Fig. S7). We then examined CF at different time points of Erdman cultures for the presence of EspR and the cytosolic marker GroEL2. EspR first appeared in the culture filtrate after 8 days of growth when it was accompanied by GroEL2, indicating that cell lysis had likely occurred (Fig. 6C).

### Discussion

The EspR protein has attracted considerable interest because of its role in the regulation of virulence in *Mtb* and the remarkable, and most unusual, property of being secreted by the very secretion system ESX-1 whose expression it controls [2]. This has led to the suggestion that a negative feedback loop modulates EspR secretion and is critical to successful infection. Studies of the three-dimensional structure of EspR and its truncated variant, EspRΔ10, together with models of DNA recognition and AFM analysis of single molecule EspR nucleoprotein complexes, indicate that EspR employs an atypical DNA recognition mechanism [10], [11]. A dimer of dimers is thought to bind to DNA via one monomer of each dimer leaving the second monomer free to contact another site. Cooperative interactions then lead to multimerization and the formation of looped structures in which EspR acts as a bridge between two separated, or even remote, sites on DNA [10], [11]. Such structures are typically formed by nucleoid-associated proteins (NAPs), like H-NS and Fis [12], [24], [25]. Collectively, these features led us to consider the possibility that EspR functions as a NAP rather than as a specific transcriptional activator of a limited number of genes required for pathogenesis.

To test this possibility, ChIP-Seq analysis was performed to assess the genome-wide distribution of EspR and to identify the sites and genes to which it binds. This resulted in the identification of at least 165 loci, often containing multiple peaks of EspR-binding, throughout the genome. Binding sites were distributed more or less evenly between intragenic and intergenic regions. The majority of the genes (∼50%) were involved in cell envelope functions. Among them were genes that contribute to ESX-1, ESX-2 and ESX-5-related activities, and others that contribute to mycobacterial virulence, such as *lipF* and the PDIM locus. Surprisingly, the latter harbored the major peak of EspR binding in the genome whereas the previously reported site preceding the *espACD* operon was less prominent (Fig. 1 and Table 1).

Confirmation of EspR binding to a selection of major sites was obtained *in vitro*, from a combination of studies performed with highly purified EspR (Fig. 3), and *in vivo*, following overexpression of the protein (Fig. 4). This resulted in the definition of a consensus sequence, TTTGC[TC][GA], that agrees well with the motif predicted previously by molecular dynamic simulations, involving computation of the binding energy of the optimal interaction of EspR with the intergenic region upstream of *espA* [10]. These predictions were consistent with the findings of DNase footprinting or EMSA studies of the same locus. Using an *in silico* approach to scan the genome sequence >1,000 potential EspR sites were found, of which 163 had been detected experimentally by ChIP-Seq. While some of the *in silico* predictions may be fortuitous, this does raise the possibility that occupancy of EspR-binding sites may vary with growth phase or physiological conditions and that more sites will be uncovered. Furthermore, the EspR-binding motif deduced here should be considered as a core sequence for high affinity nucleation sites from where cooperative binding between EspR dimers can initiate and extend to form long oligomers and hence reach more distant sites [10]. The number of such sites and the distance between them most probably enables EspR to structure the chromosome.

The number of EspR molecules per cell was estimated by quantitative Western blotting at different stages of the growth cycle. There was a steady increase in concentration until ∼100,000 molecules/cell were found at day 5. These levels are about 30-fold higher than those of well-characterized transcriptional activators, like Fnr in *E. coli*, but are comparable to levels of major NAPs, such as Fis or HU, during the exponential growth phase of *E. coli* [13], [26]. The intracellular EspR concentration is clearly in excess of that required to occupy all the experimentally detected (582) or computer predicted (1026) binding sites.

The results of subcellular fractionation of *Mtb* H37Rv cells from mid-log phase indicate that EspR is predominantly a cytosolic protein although it can be found attached to cell membrane-bound DNA, a trait of the nucleoid. Prior treatment of this fraction with DNase I releases most of the EspR to the cytosol (Fig. 6B). In contrast to the findings of a previous report [2], we were unable to detect the secreted form of EspR in the culture filtrates of ESX-1 proficient and deficient strains of H37Rv nor in the Erdman strains at early time points. Since EspR is a relatively abundant protein and only found in the culture filtrate together with the cytosolic marker GroEL2, we conclude that it is released via cell lysis rather than secretion mediated by ESX-1.

EspR seemingly acts as an activator or a repressor depending on its binding position relative to the genes it controls. EspR production appears to be autoregulated as the protein binds to its own promoter region and downregulates expression in certain conditions (Fig. 4C). Interaction at the *espR* regulatory site occurs in a contrasting manner to that seen at the *espACD* locus where there are three prominent binding sites situated far upstream of the promoter (Fig. 1). Interestingly, some tubercle bacilli, including *M. bovis* and *M. microti*, have incurred the RD8 deletion in this region of the genome [27] that completely removes the three major EspR-binding sites. Two EspR-binding sites flank the *espR* promoter thus evoking an autoregulatory mechanism whereby EspR forms a loop at the promoter that either occludes RNA polymerase (RNAP) or traps RNAP that has already bound. In ChIP-Seq experiments performed with RNAP-antibodies, a major polymerase binding site was localized (data not shown) that partially overlaps EspR peak **a** (Fig. 3), consistent with the promoter prediction from 5′ RACE.

Loss of EspR strongly attenuates *Mtb* [2], suggesting that this is due to reduced functioning of the ESX-1 system as a result of insufficient EspA levels. However, in light of the present findings, this appears to be an oversimplification as expression of the genes for several other known virulence determinants are clearly subject to EspR regulation. Foremost among these is a major locus that encodes an enzyme system required for synthesis of PDIM, and in some strains PGL, both of which contribute extensively to virulence [16], [17]. Another enzyme that has an important role in pathogenesis is the lipase, LipF [16], which has been implicated in modification of the mycobacterial cell wall as an adaptive response to acid damage [28]. LipF is also thought to degrade host lipids during infection [16]. The EspR binding site (Table 1) located far upstream of the *lipF* coding sequence overlaps the previously identified 59 bp acid-inducible promoter region, situated 515 bp from the start codon [28]. Occlusion of this site by EspR would therefore explain the observed repression of *lipF* transcription (Fig. 4D). The *lipF* gene, together with a number of other EspR gene targets like *fadD26* and *espA*, is also regulated by PhoP [29], [30] and CRP [31], frequently with opposite effects on transcription.

Regulation of transcription orchestrated by EspR seems to occur at two levels. EspR binding at promoter regions, as in the case of *espR* or *lipF*, resembles global transcriptional regulators where repression of transcription stems from occlusion of RNAP whereas activation of transcription occurs via favorable interaction with RNAP and/or other proteins. On the other hand, our genome-wide analysis revealed that more than half of the EspR-binding sites are intragenic and this refutes, at least partially, the hypothesis that EspR acts as a transcription factor *per se*. Moreover, EspR overexpression had little effect on some major EspR-bound genes (Fig. 4D), suggesting that EspR-binding does not necessarily affect transcription locally but rather serves as anchoring points to organize chromosome domains. NAPs with DNA-bridging activity, such as EspR, are often located at the boundaries of chromosomal domain loops [13] where they control gene expression in a temporal or spatial manner. In many bacteria, NAP expression levels are dependent on the growth phase [24]. This is true of *Mtb* since low EspR levels were detected at early- and mid-log phase compared to stationary phase, and premature conditional overexpression causes growth to slow down. The interplay between different NAPs alters chromosome structure and organization thereby influencing patterns of gene expression in a temporal manner.

Lsr2 is a DNA-bridging protein that also performs NAP functions in *Mtb* by recognizing AT-rich and xenogeneic regions. Binding sites of Lsr2 in *Mtb* have been mapped by Gordon *et al.* using ChIP-on-chip technology [32]. Comparison of the Lsr2 ChIP-on-chip and EspR ChIP-Seq results showed that 77% of the genes in the EspR regulon are also likely recognized by Lsr2 [32] owing to an extensive overlap between their repertoires (Fig. S2). For example, all three genes significantly upregulated upon EspR overexpression (Fig. 4D) also bind Lsr2 and major ChIP-Seq peaks of EspR are located close to Lsr2 binding sites in the ChIP-on-chip enriched regions (Fig. S2).

While Lsr2 and EspR are both subject to autoregulation there is no evidence for cross-regulation and Lsr2 seems to impact many more regions. Lsr2 has an N-terminal dimerization domain and a C-terminal DNA-binding domain whereas in EspR the opposite configuration exists. A further difference lies in the DNA recognition mechanisms since Lsr2 interacts with the minor groove while EspR binding is predicted to occur via the major groove. Together, this suggests that EspR and Lsr2 may control gene expression, including that of many cell wall functions, in a divergent manner with EspR possibly replacing Lsr2 at certain sites and vice-versa. It is striking that in all sequenced mycobacterial genomes *espR* is very close to the *hns* gene encoding another NAP [33] and this may also indicate functional interplay. These hypotheses can be tested experimentally by using the corresponding antibodies to perform ChIP-Seq experiments on *Mtb* strains at different stages in the growth cycle to localize their binding sites. The contribution of other global regulators that intersect with the EspR regulon, like PhoP and CRP, should also be examined. A regulatory scheme is emerging in which growth of *Mtb*, and hence pathogenesis, is controlled by chromosome remodeling, effected by different NAPs, thereby resulting in pleiotropic regulation of gene expression. EspR may thus play a central role in regulating virulence gene expression analagous to that of H-NS in enteropathogenic bacteria [12], [13].

## Materials and Methods

### Bacterial strains and culture conditions

*Mycobacterium tuberculosis* (*Mtb*) H37Rv and Erdman were grown in 7H9 Broth (Difco) supplemented with 0.2% glycerol, Middlebrook albumin-dextrose-catalase (ADC) enrichment and 0.05% Tween 80 or on solid Middlebrook 7H11 medium (Difco) supplemented with oleic acid-albumin-dextrose-catalase (OADC). *Mtb* cells prepared for secretion analysis and cell fractionation were grown in Sauton liquid medium. *Escherichia coli* BL21(DE3) was used for expression of His_9_-tagged EspR as described [10].

### ChIP-Seq

Chromatin immunoprecipitation was performed essentially as previously described [34] but with some variations. Briefly, *Mtb* H37Rv cultures (50 ml) grown to OD_600_ 0.4–0.6 were treated with formaldehyde (final concentration 1%) for 10 min at 37°C. Cross-linking was quenched by addition of glycine to a final concentration of 125 mM. Harvested cells were washed twice with Tris-buffered saline (20 mM Tris-HCl pH 7.5, 150 mM NaCl), re-suspended in 600 µl Immunoprecipitation (IP) buffer (50 mM Hepes-KOH pH 7.5, 150 mM NaCl, 1 mM EDTA, 1% Triton X-100, 0.1% sodium deoxycholate, 0.1% SDS, mini-protease inhibitor cocktail (Roche)) and sonicated to shear DNA to an average size of 100–500 bp. Insoluble cellular debris was removed by centrifugation and the supernatant used as input sample in IP experiments. 300 µl of input was incubated with either no antibody (mock-IP) or 20 µl of serum containing rat anti-EspR polyclonal antibodies (kindly generated by Ida Rosenkrands) and then with 50 µl of Dynabeads sheep anti-rat IgG (Dynal Biotech) pre-saturated with 0.1 mg/ml salmon sperm DNA and 1 mg/ml Bovine Serum Albumin (BSA) in IP buffer. Washing and crosslink reversal of protein-DNA complexes, as well as purification of the resulting DNA was carried out as previously described [34]. Prior to sequencing, DNA fragment sizes were checked and enrichment was verified by gene-specific quantitative PCR (ChIP-qPCR).

### EspR ChIP-Seq library construction and sequencing

DNA fragments (150 to 250 bp) were selected for library construction and sequencing libraries prepared using the ChIP-Seq Sample Preparation Kit (Illumina; San Diego, California, USA; Cat. No. IP-102-1001) according to the protocol supplied with the reagents. Prior and post library construction, chromatin immunoprecipitation products were quantified using the Qubit fluorometer (Invitrogen; Carlsbad, California, USA). One lane of each library was sequenced on the Illumina Genome Analyzer IIx using the Single-Read Cluster Generation Kit v4 and 36 Cycle Sequencing Kit v4. Data were processed using the Illumina Pipeline Software v1.60.

### ChIP-Seq data analysis

ChIP-Seq experiments, with two independent mid-log phase cultures, generated 25.6 and 22 million reads, of which 95% could be successfully mapped to the *Mtb* H37Rv genome (NCBI accession NC_000962.2) using Bowtie [35] allowing up to 3 mismatches and up to 10 hits per read. As a control, input DNA was also sequenced and mapped to the *Mtb* H37Rv genome to identify sequencing artifacts and calculate enrichment values. Since comparison of the two ChIP-Seq datasets showed excellent correlation in binding signals (Fig. S1), sequenced DNA reads from both experiments were pooled together. In order to estimate the binding location in the enriched regions, we used a deconvolution algorithm that models the expected tag distribution on both strands [36]. Based on the deconvoluted profile, a score was calculated for each peak that was proportional to the read density in the peak. Considering the high genome coverage, only peaks with more than 600 reads per position were selected. Reads mapped to the forward and reverse strands were shifted (by 80 bp) and merged together to generate single peak profiles, which were visualized on the UCSC Genome Browser database [37]. Annotation of the peaks was performed using the *ChipPeakAnno* package from Bioconductor [38]. The read count at each binding region (400 bp width) was determined as the total number of reads mapping to the region divided by the length of the region normalized to the total number of mapped reads across the whole genome. The enrichment at each locus was obtained as the ratio of the average read count from the ChIP sample (from two datasets) to the read count from the input DNA sample. Peaks with an enrichment ratio lower than 1.5 were filtered out. The ChIP-Seq data files have been deposited in NCBI's Gene Expression Omnibus [39] and can be accessed through GEO Series accession number GSE35149. Lsr2 data were retrieved from this database (accession number GSE18652).

### Motif detection

Sequences of 102 bp covering the center of the 582 peaks that fulfilled the peak selection criteria were extracted and used for motif analysis using MEME [19] with motif occurrence set as “zero or one per sequence”, minimum width as 5 and other parameters as default. Since many EspR binding sites are found within the highly repetitive sequences belonging to *pe* and *ppe* genes, the initial motif returned by MEME was biased and reflected a *pe*-*ppe* motif. Thus, from the 582 sequences, those belonging to the *pe* and *ppe* categories were excluded, leaving 416 sequences that were reanalyzed using MEME. This led to the identification of an unbiased overrepresented motif shown in Fig. 2. This motif was further used in FIMO (Find Individual Motif Occurences) from the MEME Suite web server [19] to search for motif occurrence in the entire set of EspR binding sequences setting the *p*-value threshold to 0.001.

### Electrophoretic mobility shift assays (EMSA) and DNase I footprinting

EspR protein was produced and purified as previously described [10] and used in EMSA, DNase I footprinting and quantitative Western Blot experiments, as well as to immunize rats. EspR-DNA gel retardation assays were performed as recently described [10] using 5′-biotin-labeled forward primers and unlabeled reverse primers in PCR reactions designed to yield DNA probes of 99 to 120 bp encompassing selected ChIP-Seq peak sequences for analysis. DNase I footprinting assay was carried out using 6-FAM-labeled probes as described in [10]. 6-FAM-labeled forward primers and unlabeled reverse primers were used to synthesize a 361 bp DNA fragment starting 208 bp upstream of the *espR* translational start by PCR. The nucleotide sequences of protected regions were determined by DNA sequencing.

### 5′ RACE

RNA was extracted from *Mtb* H37Rv cells in the mid-log phase. 5′ RACE was performed as previously described [34] using the 5′/3′ RACE kit (2nd Generation, Roche) and primers specific for *espA* and *espR* for 5′-end mapping (see Table S1).

### Inducible overexpression of EspR in *Mtb* H37Rv

Construction of the pMYespR vector for pristinamycin IA-inducible overexpression of *espR* was undertaken as previously described [21]. Plasmids pMYespR and the control pMY769 were transformed into H37Rv strain by electroporation, clones were selected on 7H11 agar plates containing 100 µg/ml spectinomycin and plasmid integration verified by PCR. The resulting H37Rv::pMYEspR strain was grown to mid-log phase (OD_600_ 0.4–0.6), diluted to OD_600_ = 0.1 (day 0) and split into two 30 ml volumes prior to addition of 0 or 2 µg/ml pristinamycin IA. Cells were harvested at day 3.

For whole cell Western blot analysis, bacteria were pelleted, washed twice in PBS containing 0.05% Tween 80 and resuspended in lysis buffer (10 mM Tris pH 7.9, 500 mM NaCl, 1 mM β-mercaptoethanol, 5% glycerol, 0.1 mM EDTA) prior to sonication (15 min at 4°C). Cell debris was then pelleted and the supernatant filter sterilized. After total protein quantification using Bradford reagent (Sigma), 10 µg of cell lysates were separated by SDS-PAGE, and specific proteins visualized by immunoblot as described below.

### Immunoblot analysis

Proteins were separated on NuPAGE Novex 4–12% bis-Tris gels (Invitrogen) and transferred to nitrocellulose membranes. Membranes were incubated in TNT blocking buffer (25 mM Tris pH 7.5, 150 mM NaCl, 0.05% Tween 20) with 5% w/v skim milk powder for 2 h prior to incubation with primary antibodies diluted in TNT with 1% BSA overnight. Membranes were washed in TNT five times, then incubated with secondary antibodies for 1 h before washing. Immunoblotting were performed with rat polyclonal anti-EspR antibodies (kindly provided by Ida Rosenkrands), mouse monoclonal anti-EsxA antibodies (Hyb 76-8), mouse monoclonal anti-GroEL2 antibodies (IT-70), rabbit polyclonal anti-EsxB and anti-SodA antibodies. All antibodies were used at a dilution 1∶1,000 except for anti-GroEL2 (1∶5,000), anti-EsxB (1∶500) and anti-SodA (1∶50). Horseradish peroxidase (HRP) conjugated rabbit polyclonal anti-rat IgG, rabbit polyclonal anti-mouse IgG or goat polyclonal anti-rabbit IgG secondary antibodies (Sigma) were used at dilutions of 1∶100,000. Secondary antibodies were visualized using chemiluminescent substrates (Sigma).

### Quantitative RT-PCR

Quantitative RT-PCR reactions were performed with the 7900HT Fast Real-Time PCR System (Applied Biosystems) with the following parameters: 50°C for 2 min, 95°C for 10 min, followed by 40 cycles of 95°C for 15 s and 60°C for 60 s. Melt curve analysis was used to confirm specific amplification for each primer pair. Threshold cycle values were determined automatically using the SDS software.

Enrichment of ChIP DNA samples was measured by quantitative RT-PCR using peak-specific primer pairs (Table S1), IP or mock-IP DNA as template and SYBR-Green master mix (Applied Biosystems). To calculate the amount of amplified DNA, standard curves were generated for each primer pair using 10-fold dilutions of input DNA as template. All reactions were done in duplicate and averaged to determine the enrichment ratio calculated as [IP DNA]/[mock-IP DNA].

For gene expression analysis, RNA was extracted with Trizol reagent (Invitrogen) and treated with DNase I (Roche) prior to generation of the cDNA template. cDNA was synthesized using the RevertAid First Strand cDNA Synthesis Kit (Fermentas) according to the manufacturer's instructions using random hexamers primer. cDNA corresponding to 10 ng of input RNA was used in each RT-PCR reaction supplemented with specific primer pairs (200 nM each) listed in Table S1 and SYBR-Green master mix (Applied Biosystems). Relative mRNA levels were calculated using the ΔΔCt method, normalizing transcript levels to *sigA* signals and are average of three biological replicates.

### Time course expression analysis

*Mtb* H37Rv cells were grown to OD_600_ 0.6 and then diluted in 100 ml with a starting OD_600_ of 0.05 (day 0). At various time points corresponding to the early-log (day 2), mid-log (day 3) and stationary (days 4 and 5) phase of growth, the number of cells was estimated from OD_600_ and culture aliquots were pelleted by centrifugation for RNA and protein analysis. For protein analysis, 400 to 100 µl cell aliquots were resuspended in a 1× NuPAGE sample buffer (Invitrogen) and boiled for 45 min. For all time points, equivalent amounts of cells were subjected to SDS-PAGE together with purified EspR protein standards (40, 30, 20, 15, 10 and 7.5 ng) and proteins detected by immunoblotting. Quantification was performed by densitometry of scanned blots using the ImageJ software. A standard curve was made from the intensity of the purified EspR bands and was shown to be linear in the 7.5 to 40 ng range. Quantification of expression levels at each time point was estimated from the standard curve after correction for small sample loading discrepancies using GroEL2 band intensity for normalization. The experiment was repeated four times and a typical immunoblot pattern of EspR expression is shown in Fig. 5A. For transcript analysis, RNA isolation and quantitative RT-PCR was performed as described above.

### EspR secretion analysis

*Mtb* starter cultures were first grown in complete 7H9 broth to late-logarithmic phase (OD_600_∼0.8–1). These were then used to inoculate Sauton's medium supplemented with 0.05% Tween-80, at starting OD_600_ of 0.1. Cells were grown to mid-log phase of growth (OD_600_∼0.6), centrifuged, washed once with phosphate buffered saline (PBS), resuspended in a volume of Sauton's medium without Tween-80 such that the OD_600_ was again ∼0.6 then grown further at 37°C with shaking. Cultures were harvested at required times post-transfer by centrifugation to obtain culture filtrates and cell pellets. Culture filtrates (CF) were filtered sequentially through 0.4 and 0.2 micron filters to remove any residual cells and concentrated in Vivaspin columns with 5-kDa molecular weight cut-off membranes (Sartorius Stedim Biotech GmbH, Goettingen, Germany). Cell lysates (CL) were prepared by resuspending cell pellets in lysis buffer (PBS containing Roche mini-protease inhibitor cocktail tablets) followed by bead beating with 100 micron glass beads and centrifuged. Total protein concentration of all preparations was determined using the BCA assays (Pierce) with BSA as the standard. Equivalent total protein concentrations of CF and CL were analyzed by SDS-PAGE and immunoblotting. For cell fractionation experiments, CL samples were split into two equivalent volumes and treated with either 50 µl PBS or 50 µl of 1,000 units/ml DNase I overnight at 4°C. CL samples were centrifuged at 200,000 g for 4 h at 4°C in a Beckman-Coulter ultracentrifuge (TLA-100.3 rotor) to obtain the cell wall/cell membrane (W/M) fraction. Supernatants containing the cytosolic (CYT) fractions were carefully recovered and the W/M fractions resuspended in PBS with 0.1% Triton X-100 and mini-protease inhibitor cocktail. Equivalent amounts of the different cell fractions were separated by SDS-PAGE and specific proteins were visualized by immunoblotting.

## Supporting Information

Figure S1 Correlation plot showing the reproducibility of EspR ChIP-Seq.
(PDF)

Figure S2 Comparison of EspR and Lsr2 binding sites on the *Mtb* genome. (A) Venn diagram showing significant overlap between the gene targets of Lsr2 [32] as obtained by ChIP-on-chip and EspR as obtained by ChIP-Seq (this study). (B–F) UCSC Genome Browser (http://genome.ucsc.edu) view of selected binding profiles as determined by ChIP-Seq for EspR (green, this study) and by ChIP-on-chip for Lsr2 (red, [32]). Shown are (B) the *rv1490* region; (C) the *rv0986*-*7*-*8* operon region; (D) the *espA*-*ephA* intergenic region; (E) the ESX-1 (*rv3864*-*3883c*) and ESX-2 (*rv3884c*-*rv3895c*) regions; and (F) the PDIM/PGL locus (*rv2928*-*rv2962c*). The GC content of *Mtb* H37Rv genome (in 20 bp windows) normalized to the median GC content (65.6%) and the gene positions are indicated below.
(PDF)

Figure S3 Analysis of the *espA* promoter. RNA extracted from *Mtb* H37Rv at mid-log phase was used for the 5′ RACE. Translational starts are highlighted in red with +1 corresponding to the first base of the *espA* open reading frame. Transcriptional start mapped by 5′ RACE is boxed and indicated by a bent arrow. −10 and −35 positions for putative sigma factor binding sites are indicated. ChIP-Seq peak sequences are highlighted in gray.
(PDF)

Figure S4 Validation of 11 EspR-binding sites selected among a wide range of scores by quantitative RT-PCR. The *sigA* and *rv0888* genes, showing no peak in EspR ChIP-Seq, were used as negative controls. Plot showing log_2_ enrichment calculated from ChIP-Seq and ChIP-qPCR experiments shows a good correlation between the output of the two experiments. The log_2_ enrichment values have also been indicated in the table below along with additional details about the peaks. The column “Peak feature” indicates where the EspR-binding site is located relative to the known gene annotation. “NA” denotes not applicable.
(PDF)

Figure S5 EMSA showing binding to the top five ChIP-Seq peak sequences related to the following genes: *fadD26*, *rv1490*, *rv2929*, *pe_pgrs19*, *espA*. A DNA fragment from within the *espA* coding region where no ChIP-Seq enrichment was observed was used as negative control.
(PDF)

Figure S6 DNase I footprint at peak “a” of the *espR* promoter. Red and black peaks represent DNA incubated without or with 10 µM EspR proteins, respectively. Both reactions were partially digested with DNase I and analysed by capillary electrophoresis in a genetic analyser (Applied Biosystems 3130xl). The corresponding sequencing reaction of the DNA fragment is shown at bottom. Regions I and II protected from DNase I digestion by EspR and regions IIaΔ10 and IIbΔ10 protected from DNase I digestion by EspRΔ10 are denoted by square brackets. Positions indicated are relative to the translational start.
(PDF)

Figure S7 Immunoblot analysis of 10 µg of culture filtrate (CF) and 5 µg of cell lysates (CL) of *Mtb* Erdman wild-type (left) and 36–72 (transposon insertion in the *pe35* promoter blocking *esxA* expression and therefore ESX-1 function [23]) (right); strains were grown for 4 days after transfer into Sauton's medium without Tween-80. GroEL2 was used as a control for autolysis, SodA as a loading control for CF and CL samples and EsxA as a control for ESX-1-dependent secretion.
(PDF)

Table S1 List of primers used in this study.
(PDF)

Table S2 List of the EspR ChIP-Seq peaks on the *Mtb* H37Rv genome sorted by score (highest to lowest). Peaks are numbered by order of appearance in the genome. The column “Feature” indicates where the EspR-binding site is located relative to the known gene annotation. The “Distance to feature” column designates the distance (in bp) from the midpoint position of the calculated peak to the closest translational start site. Values in the “Score” column are arbitrary units proportional to the peak density. The “Enrichment” column represents the ratio between the average ChIP-Seq read count at a particular locus (400 bp) and the input DNA read count at that locus.
(XLS)
