# Supplementary figures and images for: Virulence Regulator EspR of Mycobacterium tuberculosis Is a Nucleoid-Associated Protein
Source: PLoS Pathog. 2012 Mar 29;8(3):e1002621. doi: 10.1371/journal.ppat.1002621 (PMC3315491; doi:10.1371/journal.ppat.1002621)

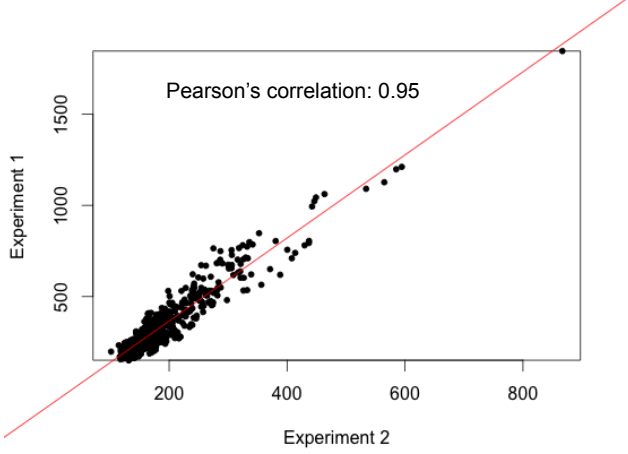

Supplement: Figure S1 — Correlation plot showing the reproducibility of EspR ChIP-Seq. (PDF) [file ppat.1002621.s001.pdf]

*fadD26*

*rv1490*

EspR [uM] 0 0.6 1.2 1.8 2.4 0 0.6 1.2 1.8 2.4

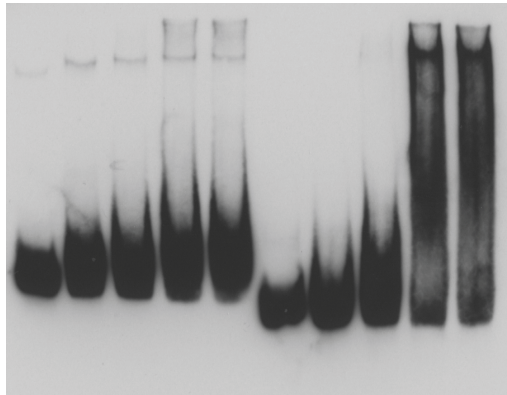

*rv2929*

*pe\_pgrs19*

EspR [uM] 0 0.6 1.2 1.8 2.4 0 0.6 1.2 1.8 2.4

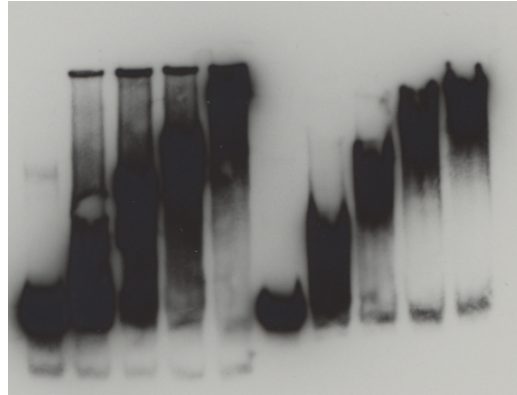

*espA*

*espA* ORF control

EspR [uM] 0 0.6 1.2 1.8 2.4 0 0.6 1.2 1.8 2.4

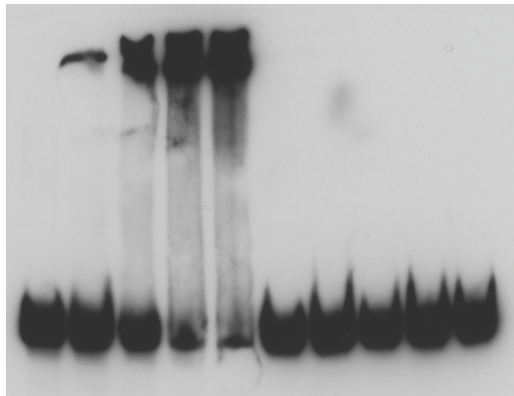

Supplement: Figure S5 — EMSA showing binding to the top five ChIP-Seq peak sequences related to the following genes: fadD26, rv1490, rv2929, pe_pgrs19, espA. A DNA fragment from within the espA coding region where no ChIP-Seq enrichment was observed was used as negative control. (PDF) [file ppat.1002621.s005.pdf]

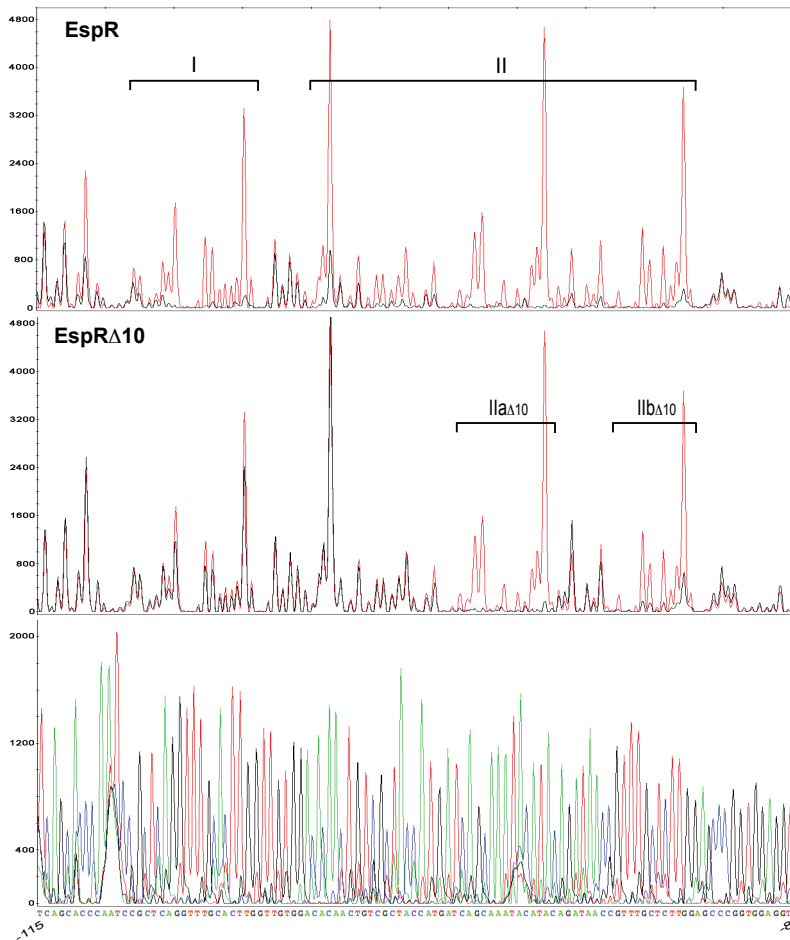

Supplement: Figure S6 — DNase I footprint at peak “a” of the espR promoter. Red and black peaks represent DNA incubated without or with 10 µM EspR proteins, respectively. Both reactions were partially digested with DNase I and analysed by capillary electrophoresis in a genetic analyser (Applied Biosystems 3130xl). The corresponding sequencing reaction of the DNA fragment is shown at bottom. Regions I and II protected from DNase I digestion by EspR and regions IIaΔ10 and IIbΔ10 protected from DNase I digestion by EspRΔ10 are denoted by square brackets. Positions indicated are relative to the translational start. (PDF) [file ppat.1002621.s006.pdf]

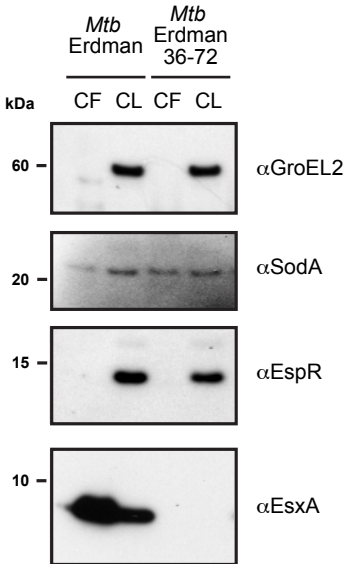

Supplement: Figure S7 — Immunoblot analysis of 10 µg of culture filtrate (CF) and 5 µg of cell lysates (CL) of Mtb Erdman wild-type (left) and 36–72 (transposon insertion in the pe35 promoter blocking esxA expression and therefore ESX-1 function [23]) (right); strains were grown for 4 days after transfer into Sauton's medium without Tween-80. GroEL2 was used as a control for autolysis, SodA as a loading control for CF and CL samples and EsxA as a control for ESX-1-dependent secretion. (PDF) [file ppat.1002621.s007.pdf]
