# Supplementary material for: Virulence Regulator EspR of Mycobacterium tuberculosis Is a Nucleoid-Associated Protein
Source: PLoS Pathog. 2012 Mar 29;8(3):e1002621. doi: 10.1371/journal.ppat.1002621 (PMC3315491; doi:10.1371/journal.ppat.1002621)
Supplement: Figure S2 — Comparison of EspR and Lsr2 binding sites on the Mtb genome. (A) Venn diagram showing significant overlap between the gene targets of Lsr2 [32] as obtained by ChIP-on-chip and EspR as obtained by ChIP-Seq (this study). (B–F) UCSC Genome Browser (http://genome.ucsc.edu) view of selected binding profiles as determined by ChIP-Seq for EspR (green, this study) and by ChIP-on-chip for Lsr2 (red, [32]). Shown are (B) the rv1490 region; (C) the rv0986-7-8 operon region; (D) the espA-ephA intergenic region; (E) the ESX-1 (rv3864-3883c) and ESX-2 (rv3884c-rv3895c) regions; and (F) the PDIM/PGL locus (rv2928-rv2962c). The GC content of Mtb H37Rv genome (in 20 bp windows) normalized to the median GC content (65.6%) and the gene positions are indicated below. (PDF) [file ppat.1002621.s002.pdf]

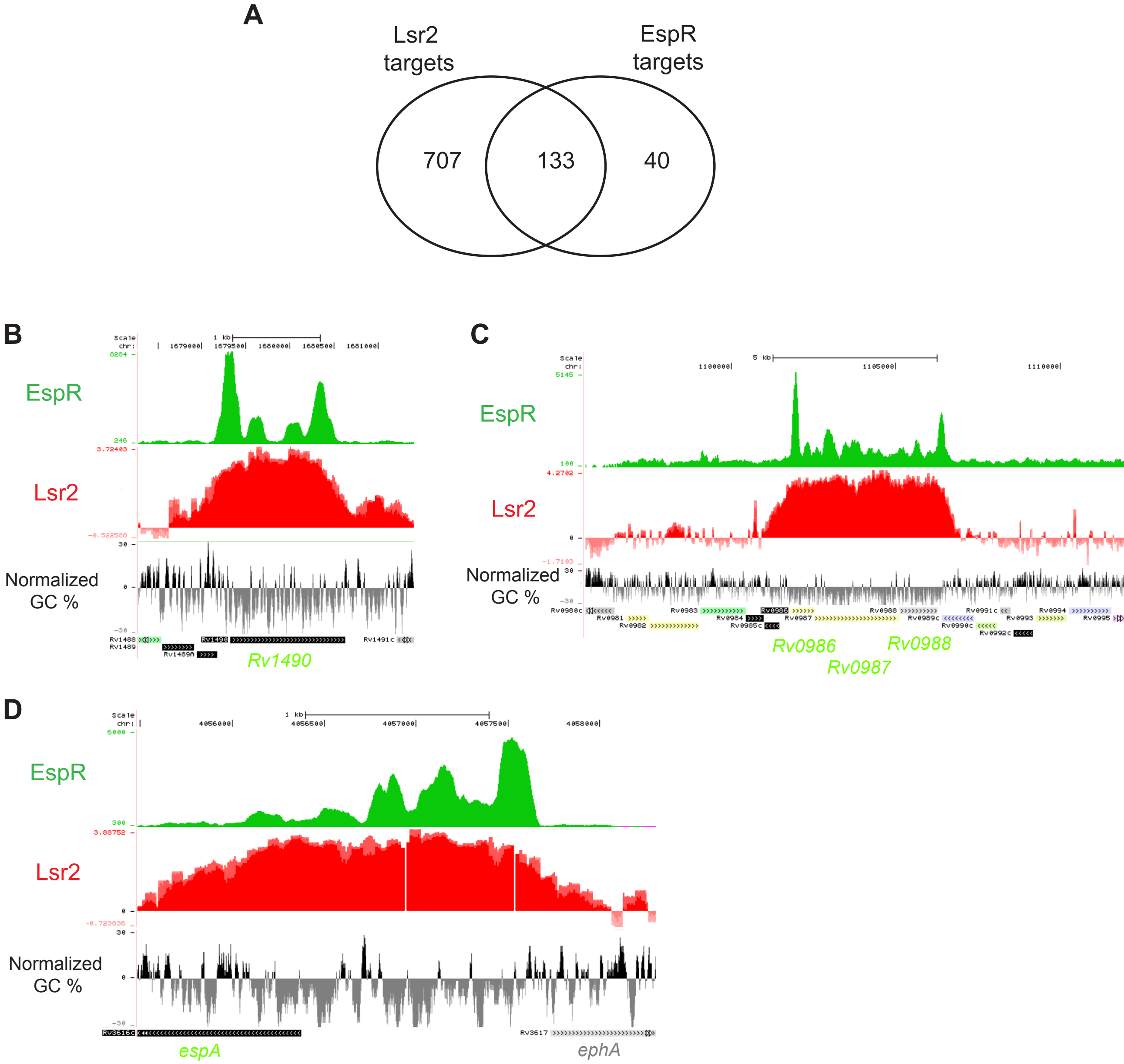
A
Lsr2 targets
EspR targets
707
133
40
B
EspR
Lsr2
Normalized GC %
Rv1490
C
EspR
Lsr2
Normalized GC %
Rv0986
Rv0987
Rv0988
D
EspR
Lsr2
Normalized GC %
espA
ephA

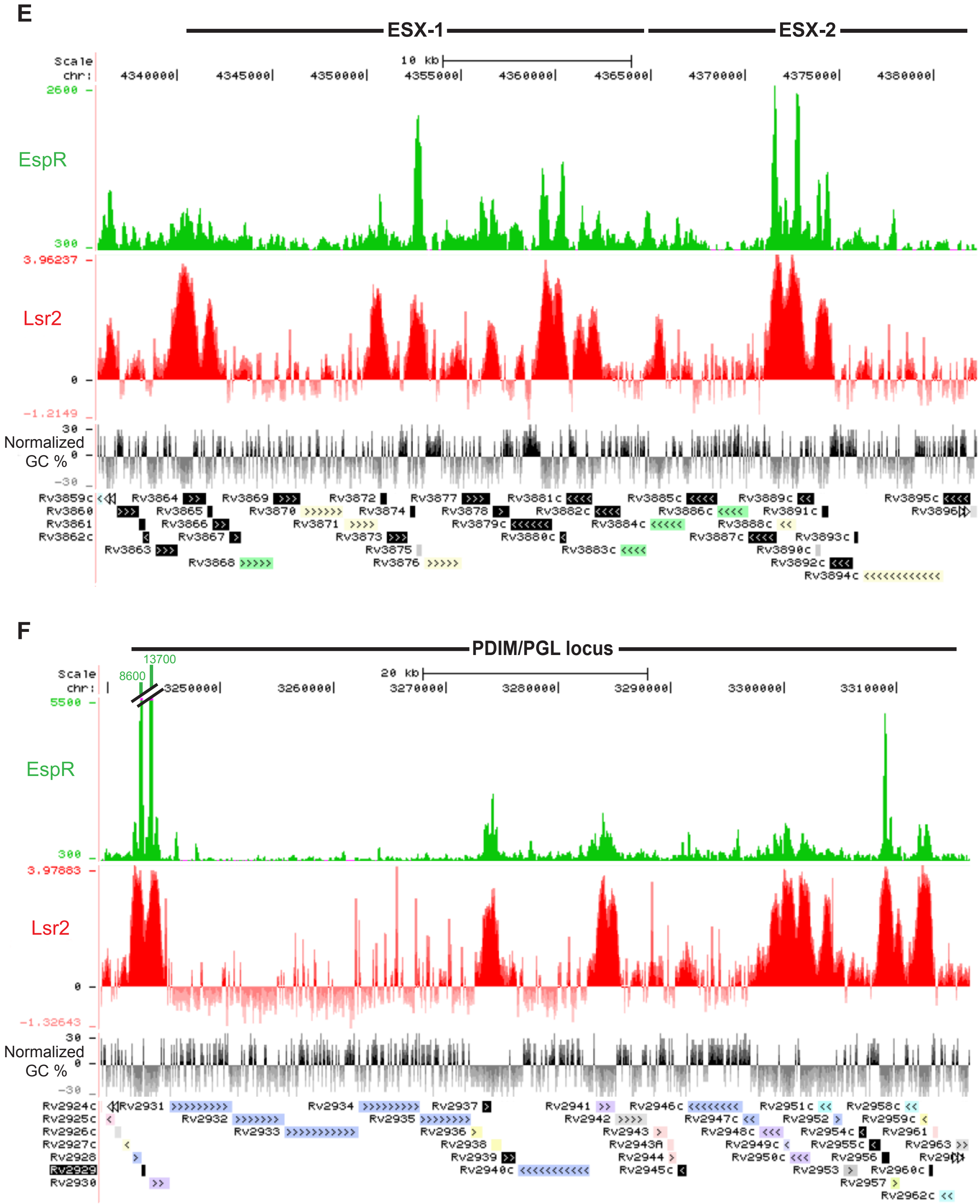
E
ESX-1
ESX-2
Scale
chr:
10 kb
4340000|
4345000|
4350000|
4355000|
4360000|
4365000|
4370000|
4375000|
4380000|
2600 -
300 _
EspR
3.96237 -
0 -
-1.2149 _
Lsr2
30 -
0 -
-30 _
Normalized GC %
Rv3859c
Rv3860
Rv3861
Rv3862c
Rv3863
Rv3864
Rv3865
Rv3866
Rv3867
Rv3868
Rv3869
Rv3870
Rv3871
Rv3872
Rv3873
Rv3874
Rv3875
Rv3876
Rv3877
Rv3878
Rv3879c
Rv3880c
Rv3881c
Rv3882c
Rv3883c
Rv3884c
Rv3885c
Rv3886c
Rv3887c
Rv3888c
Rv3889c
Rv3890c
Rv3891c
Rv3892c
Rv3893c
Rv3894c
Rv3895c
Rv3896
F
PDIM/PGL locus
8600
13700
Scale
chr:
20 kb
3250000|
3260000|
3270000|
3280000|
3290000|
3300000|
3310000|
5500 -
300 _
EspR
3.97883 -
0 -
-1.32643 _
Lsr2
30 -
0 -
-30 _
Normalized GC %
Rv2924c
Rv2925c
Rv2926c
Rv2927c
Rv2928
Rv2929
Rv2930
Rv2931
Rv2932
Rv2933
Rv2934
Rv2935
Rv2936
Rv2937
Rv2938
Rv2939
Rv2940c
Rv2941
Rv2942
Rv2943
Rv2943A
Rv2944
Rv2945c
Rv2946c
Rv2947c
Rv2948c
Rv2949c
Rv2950c
Rv2951c
Rv2952
Rv2953
Rv2954c
Rv2955c
Rv2956
Rv2957
Rv2958c
Rv2959c
Rv2960c
Rv2961
Rv2962c
Rv2963
