# Supplementary material for: Virulence Regulator EspR of Mycobacterium tuberculosis Is a Nucleoid-Associated Protein
Source: PLoS Pathog. 2012 Mar 29;8(3):e1002621. doi: 10.1371/journal.ppat.1002621 (PMC3315491; doi:10.1371/journal.ppat.1002621)
Supplement: Figure S3 — Analysis of the espA promoter. RNA extracted from Mtb H37Rv at mid-log phase was used for the 5′ RACE. Translational starts are highlighted in red with +1 corresponding to the first base of the espA open reading frame. Transcriptional start mapped by 5′ RACE is boxed and indicated by a bent arrow. −10 and −35 positions for putative sigma factor binding sites are indicated. ChIP-Seq peak sequences are highlighted in gray. (PDF) [file ppat.1002621.s003.pdf]

ephA (rv3617)

```
-1360 CATCGTCTCACCAAATCACACCGGGCACCGGCCTGAGAAGGGCTTGGGGAGCAGCCAGAGGCGATTGTCGCGGGTGCTGC
-1360 GTAGCAGAGTGGTTTAGTGTGGCCCGTGGCCGGACTCTTCCCGAACCCCTCGTCGGTCTCCGCTAACAGCGCCCACGACG

-1280 CGCGCATCATTGATCGGCCGGCCGGACCAGTCGGGCCTCCCTTGACGTCCGGATCGCACTTCCTGTGCAGCTGGCATGGC
-1280 GCGCGTAGTAACTAGCCGGCCGGCCTGGTCAGCCCGGAGGGAACTGCAGGCCTAGCGTGAAGGACACGTCGACCGTACCG

-1200 TACAGCTCACAGTGACTGCCCCACGATTGCCGGCCAGGTCCAGTTCAAATTCCGGTGAATTCGCGGACAAAAGCAGCAGG
-1200 ATGTCGAGTGTCACTGACGGGGTGCTAACGGCCGGTCCAGGTCAAGTTTAAGGCCACTTAAGCGCCTGTTTTCGTCGTCC

-1120 TCAACCAACCGCAGTCAGTCGAGGGTCCCAAACGTGAGCCAATCGGTGAAATGGCTTGCTGCAGTGACACCGGTCACAGG
-1120 AGTTGGTTGGCGTCAGTCAGCTCCCAGGGTTTGCACTCGGTTAGCCACTTTACCGAACGACGTCACTGTGGCCAGTGTCC

-1040 CTTAGCCGACAGCACCGGAATAGCTCAGGCGGGCTATAGAGTCCTATAGAAACATTTGCTGATAGAATTAACCGCTGTCT
-1040 GAATCGGCTGTCGTGGCCTTATCGAGTCCGCCCGATATCTCAGGATATCTTTGTAAACGACTATCTTAATTGGCGACAGA

 -960 TGGCGTGATCTTGATACGGCTCGCCGTGCGACCGGTTGGCTCAGTAGCTGACCACCATGTAACCCATCCTCGGCAGGTGT
 -960 ACCGCACTAGAACTATGCCGAGCGGCACGCTGGCCAACCGAGTCATCGACTGGTGGTACATTGGGTAGGAGCCGTCCACA

 -880 CTACTAAGGCGAGACACCGCATTGGTGGGGCTGCATCGCAAATCGGTCCGAGCATGTAGCACTGCCGTTATCCCGGGATA
 -880 GATGATTCCGCTCTGTGGCGTAACCACCCCGACGTAGCGTTTAGCCAGGCTCGTACATCGTGACGGCAATAGGGCCCTAT

 -800 GCAAACCACCCGGAACCAGGGCTATCCCAGTCGCTCTCCGACGGAGGCCGTTTCGCTTTCCGTTGCCCGATAACTCCCGA
 -800 CGTTTGGTGGGCCTTGGTCCCGATAGGGTCAGCGAGAGGCTGCCTCCGGCAAAGCGAAAGGCAACGGGCTATTGAGGGCT

 -720 GTGGATATCGGCGTTATCAGATTCAGGCTTTTCTTCGCAAGGTACCGGTGTTCGCTATATTCGGATATCTCGGACGGATA
 -720 CACCTATAGCCGCAATAGTCTAAGTCCGAAAAGAAGCGTTCCATGGCCACAAGCGATATAAGCCTATAGAGCCTGCCTAT

 -640 ATTACTAAAACTTCAGTGGTTTAGATAAGGCCGCCGCAATACTTCGCCGATCTTGCCGAGCGCAACGGATTTCCATCGTC
 -640 TAATGATTTTGAAGTCACCAAATCTATTCCGGCGGCGTTATGAAGCGGCTAGAACGGCTCGCGTTGCCTAAAGGTAGCAG

 -560 GGTTTTCGTCGCCTTATCAAACATGATCGGAGATAATGACAGATCGGCCTAGCTAGGTGTTTAGCGGACGCGATTTAGGA
 -560 CCAAAAGCAGCGGAATAGTTTGTACTAGCCTCTATTACTGTCTAGCCGGATCGATCCACAAATCGCCTGCGCTAAATCCT

 -480 CAACCGAGATTTGCTTTGCCTCGCAACCATGAGAGCGCCCCGCTTCGACGCCGAATCGGGTGAGTGATGGTGGGTTAGCA
 -480 GTTGGCTCTAAACGAAACGGAGCGTTGGTACTCTCGCGGGGCGAAGCTGCGGCTTAGCCCACTCACTACCACCCAATCGT

 -400 CAGCCCTGATTGCGCCACCGGCGAGGTGATTGTGCCCGCCACGAGGCCGCCGCCGGCTAGCCCCATGAGCACACTATATA
 -400 GTCGGGACTAACGCGGTGGCCGCTCCACTAACACGGGCGGTGCTCCGGCGGCGGCCGATCGGGGTACTCGTGTGATATAT

 -320 GACTCTCCTGCAACAGATCTCATACCGATCGAAGGCGAAGCGCAGGCATCGACGTCGGAGACACTGCCTTGGGATCGCGC
 -320 CTGAGAGGACGTTGTCTAGAGTATGGCTAGCTTCCGCTTCGCGTCCGTAGCTGCAGCCTCTGTGACGGAACCCTAGCGCG

 -240 CGCCTACACGGCGGTTGGCGCATTGTCGCAGCGCAGTTGCAGGAGGGCAAATGTGCGCAGACGATGTAGTCGACAACAAG
 -240 GCGGATGTGCCGCCAACCGCGTAACAGCGTCGCGTCAACGTCCTCCCGTTTACACGCGTCTGCTACATCAGCTGTTGTTC
                                                                                -35
 -160 TGAACATGCCGTCTTCACGAACTCAAAACTGACGATCTGCTTAGCATGAAAAAAACTGTTGACATCGGCCAAGCATGACA
 -160 ACTTGTACGGCAGAAGTGCTTGAGTTTTGACTGCTAGACGAATCGTACTTTTTTTGACAACTGTAGCCGGTTCGTACTGT
          -10
  -80 GCCAGACTGTAGGCCCTACGCGTGCAATGCAGAACCAAGGCTATGCATGGAATCGACGACCGTTGAGATAGGCGGCAGGC
  -80 CGGTCTGACATCCGGGATGCGCACGTTACGTCTTGGTTCCGATACGTACCTTAGCTGCTGGCAACTCTATCCGCCGTCCG

    1 ATG
    1 TAC
```

espA (rv3616c)
