# Supplementary material for: Virulence Regulator EspR of Mycobacterium tuberculosis Is a Nucleoid-Associated Protein
Source: PLoS Pathog. 2012 Mar 29;8(3):e1002621. doi: 10.1371/journal.ppat.1002621 (PMC3315491; doi:10.1371/journal.ppat.1002621)
Supplement: Figure S4 — Validation of 11 EspR-binding sites selected among a wide range of scores by quantitative RT-PCR. The sigA and rv0888 genes, showing no peak in EspR ChIP-Seq, were used as negative controls. Plot showing log2 enrichment calculated from ChIP-Seq and ChIP-qPCR experiments shows a good correlation between the output of the two experiments. The log2 enrichment values have also been indicated in the table below along with additional details about the peaks. The column “Peak feature” indicates where the EspR-binding site is located relative to the known gene annotation. “NA” denotes not applicable. (PDF) [file ppat.1002621.s004.pdf]

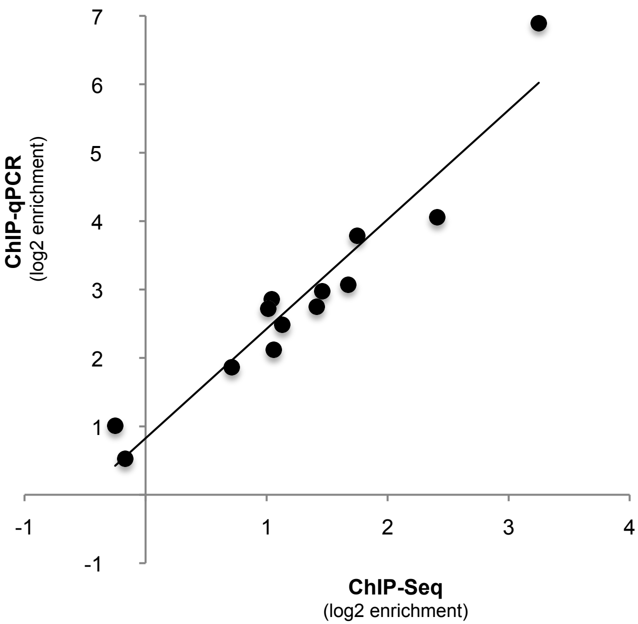

Rv number	Gene name	Peak number	Peak start	Peak end	Peak feature	ChIP-Seq Score	ChIP-Seq (log2 enrich.)	ChIP-qPCR (log2 enrich.)
Rv0888	*rv0888*	NA	NA	NA	NA	NA	-0.2	0.5
Rv2703	*sigA*	NA	NA	NA	NA	NA	-0.3	1.0
Rv2930	*fadD26*	395	3243769	3243870	inside	1,267,869	3.2	6.9
Rv3616c	*espA*	563	4056792	4056893	upstream	302,625	1.7	3.8
Rv2336	*rv2336*	340	2610813	2610914	overlapStart	301,250	1.7	3.1
Rv0304c	*ppe5*	61	372558	372659	inside	244,486	2.4	4.1
Rv3888c	*3888c*	620	4372704	4372805	overlapStart	194,815	1.0	2.9
Rv3343c	*ppe54*	506	3734612	3734713	inside	172,539	1.4	2.7
Rv3875	*esxA*	609	4352602	4352703	overlapStart	118,926	1.5	3.0
Rv1795	*eccD5*	294	2032054	2032155	upstream	116,034	1.1	2.5
Rv0507	*mmpL2*	142	597756	597857	inside	111,626	0.7	1.9
Rv3849	*espR*	604	4323381	4323482	upstream	84,752	1.1	2.1
Rv1075c	*1075c*	197	1200461	1200562	upstream	58,258	1.0	2.7
