# Supplementary material for: Virulence Regulator EspR of Mycobacterium tuberculosis Is a Nucleoid-Associated Protein
Source: PLoS Pathog. 2012 Mar 29;8(3):e1002621. doi: 10.1371/journal.ppat.1002621 (PMC3315491; doi:10.1371/journal.ppat.1002621)
Supplement: Table S1 — List of primers used in this study. (PDF) [file ppat.1002621.s008.pdf]

Table S1. Primers used in this study

Purpose	Name	Sequence (5'-3')
**ChIP-qPCR**	0888+3for	GGA TTA CGC CAA ACG CAT C
	0888+269rev	AAG TCG ATC AGG GCA TTG AG
	CS-057-sigAF	AAA CAG ATC GGC AAG GTA GC
	CS-058-sigAR	CTG GAT CAG GTC GAG AAA CG
	fadD26+110for	TTG CTG ACA GCT TGA CTT GG
	fadD26+371rev	ACG GAC GAA GTC GTG AGA AT
	CS-159-espAF	GTT TAG CGG ACG CGA TTT AG
	CS-160-espAR	TGC TAA CCC ACC ATC ACT CA
	BB-174-2336F	GTC AAA TCC GTC ACG AAC CT
	BB-175-2336R	GGT TCC CAC ACC AGT TGT TT
	ppe5+4for	AAC CTG GTC TCC ACA ACG TC
	ppe5+275rev	AAG TTA AAG CCG CCG AGA CT
	BB-186-3888cF	GAC GTT ACC AGG TCC TCT CG
	BB-187-3888cR	CGG CTC TTT GAG TGC ATT AG
	BB-172-ppe54F	ACT CAG TTG TCG GGG ATG TT
	BB-173-ppe54R	CGA ACC CAG TCA GAT TGT CC
	BB-170-esxAF	CCA AGA AGC AGC CAA TAA GC
	BB-171-esxAR	GAC GTG ACA TTT CCC TGG AT
	BB-168-eccD5F	AAG GCA AGG TCA ACC ACA AC
	BB-169-eccD5R	CAG CTA CTG CGG TCA TCG T
	BB-182-mmpL2F	AGT ATC GCT AAG GTC ACC GC
	BB-183-mmpL2R	ATG AGA ACG AGC AGG TTG GT
	espRpro196for	TGA AAT CCG TTC GTC GTG TG
	espRpro53rev	TGA TCA TGG TAG CGA CAG TTG TG
	BB-178-1075cF	TGA GCC GGA TAC CTT ACT GG
	BB-179-1075cR	CGT GGA TAG CTG ACG AAC AA
**EMSA probes PCR**	bio_espRpro106for	biotin-AAT CCG CTC AGG TTT GCA CTT G
	espRpro+6rev	GCT CAT CGA TGT GAC CTC CAC
	bio_PespR-374for	biotin-TCG CCT GAC GAG CGC TGT
	PespR-264rev	TCA GGA TGA CCG TGT CGG GC
	BiofadD26+56for	biotin-AGC CTG ACA GCA CTG CAT ATA C
	fadD26+175rev	ACT TGA GTT CTT CAG CAA TGA TGC
	Bio2929-60for	biotin-ACC TCA ACG ACA ATT TGC CAG
	2929+43rev	ATC TTC GGC CAG CTA CAT CC
	Bio1490-51for	biotin-TGC GTC GGT TAG TCT CTT TTT CG
	1490+51rev	AGT GTC GCT TGG TCG GCA
	BioPepgrs19-73for	biotin-GAA AAC CGC CCC AGT TGC
	Pepgrs19+29rev	TGC GAC GGA GAC ACC AAC
	bio_PespA-1206for	biotin-CAT GGC TAC AGC TCA CAG TGA CTG
	PespA-1108rev	TGC GGT TGG TTG ACC TGC TG
	bio_espA+541for	biotin-ATC ATT TCG GAT GTG GCG GAC
	espA+640rev	GCT TGT CCC AAA GCT CTT TCA GG
**Footprinting probes PCR**	6FAM_PespR-204for	6FAM-GAT AAC CTG TGA AAT CCG TTC GTC G
	espR+157rev	GGT TTC CTG AGC GTA GCT GTG
**5' RACE**	CS-132-espAR2	GAA GTA CTC TAG TGA GGA GTA AAG GAT ACC
	CS-133-espAR3	CCG AAC CTA ACC AGC CAT CAC C
	CS-135-espRR2	CCT GAG CGT AGC TGT GAT AGG TAG G
	CS-136-espRR3	GCG GAA GAA GTT GGC CAG G
**pMYespR vector cloning**	espR_ Pptr_rev1	GGC AGC GAA CGT CGT GCT CAT TCA GGC TCC TTG TAC GGT
	Pptr-H1	ATG TTT AAG CTT CCA TCC TGA CGG ATG GCC TTA CGA GTT C
	espR_Pptr_for2	ACC GTA CAA GGA GCC TGA ATG AGC ACG ACG TTC GCT GCC
	espR_Pptr_H2_rev2	TTT TAA GCT TCT AAG CGT CGA TCC CTT CGG CA

**qRT-PCR for gene expression**	espR+32for	GCC TGT TCG ACA CGG TTT AT
	espR+261rev	TTC CTT GTC GAG CTT TTC GT
	Pptr_espR-31for	TAT CTT CCC GTA CAC CGT ACA AG
	espR+155rev	TTT CCT GAG CGT AGC TGT GA
	PespR-136for	CTT CGT TGA CCC CTC ACA AC
	PespR-19rev	GCT CCA AGA GCA AAC GGT TA
	espA+2for	TGA GCA GAG CGT TCA TCA TC
	espA+257rev	CTG ATG AGC TGA CGA TCG AG
	ephA+216for	GAT CGA GGC CTA CGA CAT TC
	ephA+449rev	AAG TTC TCC CCA AAC CTG CT
	2929+84for	CAT TCG CTG GAC ATT TGC T
	2929+306rev	AAG AAC CGA GGT CAG AGT GC
	fadD26+110for	TTG CTG ACA GCT TGA CTT GG
	fadD26+371rev	ACG GAC GAA GTC GTG AGA AT
	1490+169for	GAA GCT GTC TTT CCG AAT GC
	1490+368rev	AGG ATA AGC CAC ACC ACG AC
	pe_pgrs19+59for	GTA TCG GTT CGG CAA TCA GC
	pe_pgrs19+312rev	GTT GAT CAA ACC CAG CAC CT
	pe_pgrs20+66for	ATC GTC GAT CAA TGC CTC A
	pe_pgrs20+313rev	CGT TGA TCA AAC CCA GCA C
	ppe24+20for	CGC CGG AGA TCA ATT CAG
	ppe24+306rev	CGT CTT GAC CGC TTC AAA CT
	0986+36for	GAG CTG GAC ATT CCG AGA AG
	0986+242rev	TCT CGC TCG GTT TTC TGA GT
	3488+31for	CTG CAC ATC CTG CAC CAC
	3488+254rev	CGA TCC TCG GTC AGT GCT
	lipF+150for	CGA CTA TCG GCT GAT CCC TA
	lipF+353rev	GGA GAG ATG GCC ACT ATT GC
	fadB2+228for	CAC CGA CCT CAA CGA CCT AT
	fadB2+443rev	ACC GGA TTG AAG AAA TGC AG
	umaA+77for	TAG ACC CCA CGA TGG CTT AC
	umaA+292rev	GAT TGC GAC TGA GCG TGA TA
	CS-057-sigAF	AAA CAG ATC GGC AAG GTA GC
	CS-058-sigAR	CTG GAT CAG GTC GAG AAA CG
